# Seventeenth Annual Meeting of the Illinois State Medical Society

**Published:** 1867-07

**Authors:** 


### SEVENTEENTH ANNUAL MEETING OF THE ILLINOIS STATE MEDICAL SOCIETY—SPRINGFIELD, JUNE 4TH AND 5TH, 1867.

The members of the Illinois State Medical Society assembled in Annual Session, in the Representatives' Hall, June 4th, 1867, at 10 o'clock A.M.

Dr. F. B. Haller, of Vandalia, President, called the Society to order, and introduced the Rev. Albert Hale, who opened the exercises with prayer.

Dr. N. Wright, Chairman of the Committee of Arrangements, then extended a cordial and appropriate welcome to the members of the Society; after which, the Assistant-Secretary, Dr. P. H. Bailhache, reported the following list of members and delegates as present:—

Drs. L. T. Hewins and T. N. Booe, of Loda, and D. L. Jewett, of Watseka, Iroquois Co.

Dr. C. Goodbrake, Clinton, DeWitt Co.

Drs. H. H. Roman, H. C. Barrell, P. J. Wardner, Justus Townsend, B. F. Stephenson, A. Trapp, Lyman B. Slater, H. B. Buck, Geo. T. Allen, B. M. Griffith, P. H. Bailhache, and H. K. Palmer, of Springfield, and N. Wright, of Chatham, Sangamon Co.

Dr. G. W. Albin, Neoga, Cumberland Co.

Drs. A. Niles, Joseph Robbins, Louis Watson, and J. T. Wilson, of Quincy, Adams Co.

Dr. F. H. VanEaton, Carrollton, Greene Co.

" M. F. DeWitt, Whitehall, "

" F. B. Haller, Vandalia, Fayette Co.

Drs. E. W. Moore, S. T. Trowbridge, J. A. W. Hostetler, and A. McBride, of Decatur, and H. N. Clark, of Niantic, Macon Co.

Dr. M. Reese, Abingdon, Knox Co.

Drs. D. Prince, G. R. Bibb, and W. S. Edgar, of Jacksonville, and John W. Craig, of Arcadia, Morgan Co.

Dr. J. T. Frazer, Howard's Point, Fayette Co.

Drs. D. W. Young and E. H. Gale, of Aurora, Kane Co.

Dr. D. S. Jenks, Plano, Kendall Co.

Drs. W. A. Knox, A. Fisher, N. S. Davis, H. A. Johnson, E. Powell, J. H. Hollister, J. Adams Allen, Moses Gunn, J. S. Hildreth, E. L. Holmes, R. N. Isham, F. O. Earle, DeLaskie Miller, C. T. Fenn, E. Ingals, and Frank W. Rielly, Chicago, Cook Co.

Dr. T. D. Washburn, Hillsboro, Montgomery Co.

Drs. E. P. Cook and J. C. Corbus, Mendota, LaSalle Co.

Dr. J. F. Potts, Peoria, Peoria Co.

" J. Little, Leroy, McLean Co.

" H. Noble, Heyworth, McLean Co.

Drs. S. W. Noble, D. O. Crist, D. L, Crist, and T. F. Worrell, Bloomington, McLean Co.

Dr. S. B. McGlumphy, Lincoln, Logan Co.

" J. S. Whitmire, Metamora, Woodford Co.

" O. Q. Herrick, Kansas, Edgar Co.

" H. W. Davis, Paris, Edgar Co.

Drs. J. O. Hamilton, J. L. White, and G. C. Lyon, Jerseyville, Jersey Co.

Dr. Wm. Massie, Grandview, Edgar Co.

" W. R. Fox, Wilmington, Will Co.

Drs. W. H. Veatch and John Ewing, Pawnee, Sangamon Co.

Dr. H. S. Hurd, Galesburg, Knox Co.

" B. K. Shurtleff, Amboy, Lee Co.

ELECTION OF MEMBERS.

The following were also elected permanent members, by unanimous vote:—

Drs. John M. West, Williamsville.
Thos. Hickman, Vandalia, Fayette Co.
B. H. Cheeny, Joliet, Will Co.
Chas. Kerr, Pawnee, Sangamon Co.
J. Sweeney, Normal, McLean Co.
A. L. Kimber, Waverly, Morgan Co.
J. P. Mathews, Carlinville, Macoupin Co.
C. L. Hart, Irving, Montgomery Co.
John Walker, Berlin, Sangamon Co.

Henry W. Boyd, Alton, Madison Co.
J. L. Millier, Springfield, Sangamon Co.
J. C. Ross, Lincoln, Logan Co.
John W. Lawrence, Carbondale, Jackson Co.
G. H. Peebles, Williamsville.

A recess of 10 minutes was taken, to enable the delegates from each county represented to select one of their own number, to constitute a committee, for nominating officers and standing committees for the ensuing year. On being called to order, the following members had been selected to constitute the Nominating Committee:—

Drs. W. S. Edgar, of Morgan Co.
Louis Watson, of Adams Co.
D. W. Young, of Kane Co.
D. S. Jenks, of Kendall Co.
J. C. Corbus, of LaSalle Co.
C. Goodbrake, of DeWitt Co.
J. F. Potts, of Peoria Co.
J. T. Frazer, of Fayette Co.
R. N. Isham, of Cook Co.
J. S. Whitmire, of Woodford Co.
M. Reese, of Knox Co.
H. B. Buck, of Sangamon Co.
S. T. Trowbridge, of Macon Co.
T. F. Worrell, of McLean Co.
G. W. Albin, of Cumberland Co.
O. Q. Herrick, of Edgar Co.
D. L. Jewett, of Iroquois Co.
S. B. McGlumphy, of Logan Co.
T. D. Washburn, of Montgomery Co.
J. L. White, of Jersey Co.
H. W. Boyd, of Madison Co.
F. H. VanEaton, of Greene Co.
B. K. Shurtleff, of Lee Co.

The Secretary read a letter from Dr. Taggert, of Cairo, explaining his inability to attend the present meeting. Also, a letter from Dr. R. G. Bogue, of Chicago, member of the

Special Committee on Deformities of the Spine and Joints, explaining the non-appearance of a report by the Committee, and asking further time, with the addition of Dr. F. O. Earle, of Chicago, to the Committee.

On motion, the request of Dr. Bogue was granted, and the Committee continued another year.

Dr. H. A. Johnson moved that the amendment to the Constitution, proposed last year, *granting to permanent members the right to vote, the same as delegates*, be taken from the table and adopted. The motion was sustained by a vote of 24 yeas and 1 nay; the members of the Nominating Committee being absent from the room.

Dr. DeLaskie Miller moved the adoption of a pending amendment to the Constitution, fixing the regular annual meetings of this Society on the *Third Tuesday in May*, in each year. The amendment was adopted by a vote of 32 yeas, 0 nays.

Dr. J. Adams Allen, Chairman of the Standing Committee on Practical Medicine and Epidemic Diseases, stated that the report consisted chiefly of two papers; one on Cholera, as it prevailed in Chicago in the summer of 1866, written by Dr. Marsh, of Chicago; another on Diseases in the Interior of the State, by Dr. L. T. Hewins, of Loda.

The first of these papers was made the special order for consideration at 2 o'clock P.M.

Dr. C. Goodbrake, Chairman of the Nominating Committee, submitted the following report:—

*For President*—Dr. S. W. NOBLE, of Bloomington.

*For Vice-Presidents*—Drs. D. W. YOUNG, of Aurora, and O. Q. HERRICK, of Kansas.

*For Treasurer*—Dr. J. H. HOLLISTER, of Chicago.

*For Next Place of Meeting*—Quincy.

On motion of Dr. H. A. Johnson, the report of the Committee was accepted, and the several candidates for office unanimously elected.

The President appointed Drs. Johnson, Miller, and Moore a committee to conduct the newly-elected officers to their places.

Drs. Noble and Young, on being conducted to their places, thanked the Society for the honor conferred on them; and the retiring President, Dr. Haller, delivered a short, but appropriate address.

The annual assessment for the year 1867, was fixed at $3.

Dr. H. A. Johnson then moved a reconsideration of the vote adopting the constitutional amendment relating to voting by permanent members.

The motion was carried, and, after a brief discussion by Drs. Johnson, Watson, Moore, Niles, Young, Worrell, and Davis, the amendment was again adopted, by a unanimous vote of 49 ayes, 0 nays.

On motion, the Society adjourned to 2 o'clock P.M.

AFTERNOON SESSION.

The Society was called to order at 2 o'clock P.M., Dr. S. W. Noble, President, in the chair.

Dr. J. Robbins, of Quincy, moved to amend the minutes of the last annual meeting, by inserting the following in the place of the fifth paragraph from the bottom of page 5 of the printed transactions:—

"A paper was presented from the Adams County Medical Society, remonstrating against the admission of the Quincy Medical Society to representation in this Society, which was referred to a special committee, consisting of S. T. Trowbridge, H. Noble, and J. M. Steele.

"The special committee, to whom was referred the communication from the Adams County Medical Society, reported the following resolution, which was adopted:—

"*Resolved*, That the so-called Quincy Medical Society is not entitled to representation in the Illinois State Medical Society."

After some discussion, the amendment proposed by Dr. Robbins was adopted, and the minutes, as amended, approved.

The special order of business being the report of the Standing Committee on Practical Medicine, Dr. J. A. Allen, Chairman of that Committee, read a paper on the Prevalence of Cholera in Chicago during the year 1866, written by Dr. W. R. Marsh of Chicago. The report was founded almost exclusively on the records of the Health-Officer of Chicago, and after some discussion by Drs. N. S. Davis, H. A. Johnson, and D. Prince, it was, on motion of Dr. P. H. Bailhache, referred to the Committee of Publication.

An invitation was received from the Mayor and city authorities of Springfield, to visit Oak Ridge Cemetery at 4 o'clock P.M., which was accepted.

Dr. J. Adams Allen presented the following resolution:—

*Resolved*, That the moderate use of ripe, but not stale or decaying, fruits is not objectionable as tending to produce cholera, but, rather, conducive to the preservation of health during the hot season.

This elicited a discussion, which was participated in by Drs. Edgar, Allen, Johnson, and Prince. While the latter was speaking, the hour fixed for the excursion to Oak Ridge arrived, and the further consideration of the subject was postponed until 9 o'clock Wednesday morning.

Notice was given that a public lecture would be given in the Hall, by Dr. J. Adams Allen, of Chicago, at 8 o'clock that evening.

The Society then adjourned to 9 o'clock A.M. of Wednesday.

WEDNESDAY, JUNE 5TH.

The Society was called to order at 9 o'clock A.M., Dr. S. W. Noble, President, in the chair.

Dr. N. Wright, in behalf of the Committee of Arrangements, reported the following list of volunteer communications, with a recommendation that they be heard in order after the reports of committees.

Tracings of the Pulse, and Shygmograph for making the same, prepared by H. A. Johnson, M.D., Prof. Chicago Med. College.

Experimental Inquiries concerning the Physiological Effects of Alcoholic Drinks. By N. S. Davis, M.D., Prof. Chicago Med. College.

Treatment of Paniform Cornea, occurring with Granular Eye-Lids. By Joseph S. Hildreth, M.D., Chicago.

Pocket Obstetric Forceps, with a description of the same. By Addison Niles, M.D., of Quincy.

A Case of Hydrophobia, with Treatment. By T. R. Higgins, M.D., of Vandalia.

Deformities of the Spine treated by Mechanical Appliances. By F. O. Earle, M.D., of Chicago.

Dr. J. H. Hollister offered the two following resolutions, which were unanimously adopted.—

*Resolved*, That the Treasurer report to the Publishing Committee, at the close of each annual meeting, the names of all those members who have been present at any one or more of the annual meetings during the *five* preceding years; or who have paid one or more annual assessments during that time; or have reported physical disability, preventing attendance at said meetings. And that only the names so reported be published in the Transactions for that year.

*Resolved*, That the Treasurer be instructed to communicate each year with all the members whose names appear in the Transactions, who have not paid their annual dues, and solicit payment of the same.

Dr. David Prince offered the following, which was adopted unanimously:—

*Resolved*, That it is with deep regret that the members of this Society contemplate the death of Daniel Brainard, M.D., Professor of Surgery in Rush Medical College; and that we unanimously accord to his memory the high appreciation due to the name of one who, by his talents and industry, has added to the sum of human knowledge, thus placing the world, as well as the profession, under obligations to perpetuate the memory of his contributions.

Dr. T. F. Worrell moved that a committee of five be appointed as a judicial committee to investigate the charges against Dr. Addison Niles, of Quincy. The motion was adopted, and the President appointed Drs. David Prince, E. W. Moore, DeLaskie Miller, O. Q. Herrick, and L. T. Hewins as said committee. And at a subsequent stage of the proceedings, the names of Drs. H. A. Johnson and Edwin Powell were added to the committee.

Dr. F. B. Haller. of Vandalia, offered the following resolution:—

*Resolved*, That we, the members of the State Medical Society, recommend that the several medical schools in this State adopt the plan of teaching recommended by the recent teachers' convention, in Cincinnati, as soon as practicable; and that we will send our pupils to the school or schools adopting such plan.

Dr. J. Adams Allen moved that the consideration of the subject be postponed until the reports from standing and special committees had been received and disposed of. Which motion was adopted.

Dr. C. Goodbrake, in behalf of the Committee on Nominations, reported the following as standing and special committees to report at the next annual meeting of the Society:—

*Committee on Practical Medicine and Epidemics*—Drs. H. A. Johnson, of Chicago; E. P. Cook, of Mendota; and Wm. Massey, of Grandview.

*Committee on Surgery*—Drs. E. Powell, of Chicago; Geo. T. Allen of Springfield; and S. T. Trowbridge, of Decatur.

*Committee on Obstetrics*—Drs. E. W. Moore, of Decatur; G. W. Albin, of Neoga; and W. A. Elder, of Bloomington.

*Committee on Drugs and Medicines*—Drs. Henry Wing, of Collinsville; W. S. Edgar, of Jacksonville; and D. S. Jenks, of Plano.

*Committee on Ophthalmology*—Drs. H. H. Roman, of Springfield; Joseph S. Hildreth, and E. L. Holmes, of Chicago.

*Committee on the Causes, Pathology, and Treatment of Cholera*—Dr. N. S. Davis, of Chicago.

*Committee on the Language of the Pulse*—Dr. J. H. Hollister, of Chicago.

*Committee on Fracture of Lower End of Radius*—Dr. David Prince, of Jacksonville.

*Committee on Conservatism in the use of Remedial Agents*—Dr. E. Ingals, of Chicago.

On motion of Dr. H. A. Johnson, Quincy was designated as the place for holding the next annual meeting of the State Medical Society.

On motion, the report of the Nominating Committee was adopted.

The resolution offered by Dr. J. A. Allen, in relation to the use of fruit during seasons when cholera is prevalent, being the special order, was taken up; Dr. Prince having the floor.

Dr. Prince advocated the resolution, and gave some interesting facts relating to the efforts to suppress the use of fruits in St. Louis during a former prevalence of cholera in that city.

Dr. W. S. Edgar thought some fruits might be used safely, while others were dangerous. Hence, he moved to amend the resolution by inserting the words "some fruits," which was lost.

Dr. E. Ingals offered the following as a substitute for the resolution of Dr. Allen, but subsequently modified his motion, so as to make it an additional resolution:—

"*Resolved*, That the diet that is ordinarily most wholesome is the proper diet to be taken during an epidemic of cholera."

After further discussion by Drs. T. F. Worrell, D. W. Young, and H. A. Johnson, Dr. N. S. Davis moved to amend the original resolution, by inserting after the word "fruits," the words "taken at the ordinary meals." He fully endorsed the position that good, ripe fruits not only did not predispose to attacks of cholera, but were positively beneficial as articles of food. But, like all other articles of food, they should be taken only at proper intervals; and, as physicians, they should not adopt any resolution that might be so construed as to encourage the eating of even ripe fruit at any and all hours of the day. The amendment was accepted without opposition.

Dr. H. A. Johnson thought it injudicious to adopt any resolution on the subject, and moved to lay the resolutions on the table. The motion was lost, by 29 yeas, 34 nays.

The resolution offered by Dr. Allen, and amended, was then adopted as follows:—

*Resolved*, That the moderate use of ripe, but not stale or decayed, fruits, taken at the ordinary meals, is not objectionable, as tending to produce cholera, but, rather, conducive to the preservation of health during the hot season.

The resolution offered by Dr. E. Ingals, was also adopted.

Dr. J. H. Hollister offered the following resolution, which was adopted:—

*Resoleed*, That a special committee of three be appointed upon the subject of Necrology, having reference to the proper preservation of statistical and other memorial records of its deceased members.

Dr. Hollister also proposed such an alteration of the Constitution as would make the Committee on Necrology a *standing* committee. Laid on the table until the next annual meeting.

Dr. J. S. Whitmire offered the following, which was adopted:

*Resolved*, That it now be made a permanent rule of this Society, that no member shall be permitted to speak a second time on any one subject, without the special permission of this Society.

Dr. E. W. Moore moved that Art. 4 of the Constitution be so amended that the term of office of the President and Vice-Presidents shall commence at the opening of the next annual meeting after their election. Laid over, under the rules.

The hearing of reports of standing committees was then resumed, and Dr. L. T. Hewins, one of the Committee on Practical Medicine, read an interesting report, which was accepted and referred to the Committee on Publication.

Dr. DeLaskie Miller, Chairman of the Committee on Obstetrics, presented an abstract of his report, which, on motion, was accepted, and the report referred to the Committee on Publication.

Dr. H. W. Davis presented a lengthy report, embodying the results of his surgical practice during his recent service in the army; which was accepted without reading and referred to the Committee on Publication.

Dr. Geo. T. Allen presented a short paper on the Radical Cure of Inguinal Hernia, with the instruments required for the operation. The paper was referred to the Committee on Publication.

Dr. Trowbridge, Chairman of the Committee appointed at the last annual meeting, to urge the enactment of suitable laws by the State Legislature, reported as follows:—

*To the Honorable President and Members of the Illinois State Medical Society:—*

The committee, to whom was entrusted the draft for the afore-mentioned law, would beg leave to report:—

That prior to the session of the General Assembly, ending the current year, there was a correspondence opened with several legislators whose opinions were favorable to the scheme, and the draft submitted to this body one year ago, at Decatur, by the Macon County Medical Society, was sent to them. There was, therefore, a very favorable element in quite a number of intelligent men of both brancues of the Legislature anxious for the passage of such an act.

About ten days after the assembling of the Legislature, a quorum of this committee met in Springfield and consulted as to a plan of procedure, and determined that the bill should make its appearance in the Senate first. It was, therefore, presented to that body by its able and zealous friend, the Hon. W. H. Cheney, of McLean Co. We had a preference that it should be referred—because of its pertinence—to the Committee on Education, which preference met the approval of the honorable senator presenting it. We consulted the members of the Committee on Education, in part, concerning the prospects of the passage of the bill, and found no opposition to it after they saw fully what it assumed to correct.

Starting it thus in the Senate, and feeling favorably impressed with the encouraging assurances which we received from various influential senators, we next consulted with many members of the House of Representatives concerning its passage through that body, should it succeed in passing through the Senate, and for a time felt sanguine of success, from the promises we received. But the golden pitcher was broken at the *fountain*. The Committee on Education tabled the bill in their room, and thus the thing was *still-born*, or rather still *unborn*, and so remained. Our honorable senator attempted to take it from the table that it might be recommitted, but it failed even in that.

There was so determined a spirit in the House that something of the kind should pass, that a bill was prosented to that body, bearing features widely differing from those which this committee was ordered to present, and considered by its friends as more conservative, which, although not killed in the committee rooms, was in the House.

It is, however, flattering, to reflect that the necessity of such a law was entertained by the Senate and House, by a larger number of men than any similar bill had ever been before. We therefore hope and expect that the intelligence of our lawmakers will so grow that to pass a statute possessing the plain-faced merit of the one which we presented will, some day soon, be the accomplishment of our continued and persistent effort. We therefore recommend that the enterprise be continued, by the appointment of a more weighty and politically influential lobbying committee.

S. T. TROWBRIDGE, M.D.
GEO. T. ALLEN, M.D.
P. H. BAILHACHE, M.D.

Dr. W. S. Edgar offered the following, which was adopted:—

*Resolved*, That a committee of five be appointed, to report a memorial and law, to be presented to the Legislature at its next regular session, for the better regulation of the practice of medicine in the State; said committee to report the result of their labors at the next meeting of the Society.

Dr. N. S. Davis, Permanent Secretary, in behalf of the Committee of Publication, presented the following report:—

When the undersigned commenced his term of office as Secretary, more than ten years since, the treasury of this Society was considerably in debt for the publication of the Transactions of the preceding year. And the receipts into the treasury have not been sufficient to liquidate the indebtedness and pay the full current expenses of publication. Hence, three alternatives have recurred to your committee at each returning year, namely:—first, to omit the publication of the Transactions annually, for want of funds; second, to publish on the individual credit of the committee, with an annually increasing balance against the treasury, which by this time would have amounted to not less than $1000; or, third, to reduce the cost of publication to the amount actually in the treasury, or nearly so. The only possible mode of accomplishing the latter alternative was, to first print the record of proceedings, reports, and papers constituting the Transactions, in the medical periodical under the control of the Secretary, and furnish extra sheets enough to make up the required number of volumes of Transactions for the use of the Society, at the cost of simple presswork, paper, and binding. In this way, from $100 to $150 could be saved to the treasury annually.

Regarding the publication of the reports and papers read before the Society, in the medical periodicals as, in itself, a benefit, by giving them a wider circulatien in the profession, your committee did not hesitate to adopt the third alternative named. This course met the uniform approval of the Society until the meeting in Decatur, June, 1867. At that meeting, which is the only one that your Secretary has failed to attend during his term of office, this subject was referred to a committee, whose report stated that it was *not* desirable to have the Transactions published in the medical journals, and recommended that 100 copies be published, independently, for the members of the Society. This report was laid on the table, without any action by the Society. Hence, the Committee of Publication were left without either an approval of their past work, or any instructions for the future.

As soon after the last meeting as the material for the Transactions had been placed in the hands of the committee, it was found that the independent publication of 100 copies of the Transactions would cost at least $260, while there was, at that time, a balance of only $1.00 in the treasury. In this dilemma, your committee resorted to the same practice as in preceding years, by which they reduced the cost of publication $111, distributed copies of the Transactions to all members who had paid the annual assessment early in November, and left the treasury free from embarassment. It is to be hoped that the increased number of members in attendance on the present annual meeting will so increase the receipts of the Treasurer, that the

Transactions of this meeting can be immediately sent to press, as an independent publication. But if, as has heretofore been the case, the amount should be found insufficient, your publication committee should not be left in the embarassing position of having neither the positive approval nor the instructions of the Society.

Immediately after the last annual meeting, the full record of proceedings was furnished for publication in both the medical journals published in our State. Subsequently, written notices were sent by the Secretary to the chairman of each committee charged with the duty of making a report at the next annual meeting. Certificates of appointment were also sent to each delegate appointed to attend the meeting of the American Medical Association for 1867.

As the duties devolving on the Publication Committee depend, for their execution, mainly upon the Permanent Secretary, the undersigned would respectfully request the Society to appoint his successor whenever they deem it advisable to do so.

Respectfully submitted, in behalf of the Com. of Publication.

N. S. DAVIS, *Permanent Secretary.*

The report was accepted, and a vote of thanks unanimously tendered to Dr. Davis, for his services in superintending the publication of the Transactions of the Society.

Dr. J. H. Hollister presented the annual report of the Treasurer, showing a balance of $2 or $3 in the treasury.

The report was accepted and referred to the Auditing Committee.

On motion, the Society adjourned to 2 o'clock P.M.

### AFTERNOON SESSION—SECOND DAY.

The meeting was called to order at 2 o'clock P.M., Dr. S. W. Noble in the chair.

Dr. D. Prince, Chairman of the Judicial Committee, made the following report, which was accepted and the committee discharged:—

*Whereas,* The claim of Dr. Addison Niles to membership in the Illinois State Medical Society rests upon the fact of his having been received as a delegate from a society which the Illinois State Medical Society has declared not entitled to representation; therefore,

*Resolved*, That the charges are not regularly before the Society.

The committee would further recommend, that inasmuch as the Quincy Medical Society was not heard in its defence last year, that the question of its right to representation be referred to a new committee, with instructions to report at the next meeting of this Society.

DAVID PRINCE,<br>

DeLASKIE MILLER,<br>

H. A. JOHNSON, } *Committee.*<br>

E. POWELL,<br>

E. W. MOORE,

Dr. J. Robbins, of Quincy, offered the following as an amendment to the report:—

*Whereas*, The claim of Dr. Addison Niles to membership in this Society rests alone on the fact that he was delegated thereto by a body (the so-called Quincy Medical Society) which this Society has declared not to be entitled to representation; therefore,

*Resolved*, That the name of Dr. Addison Niles be stricken from the roll of members.

On motion of Dr. H. Noble, the amendment was laid on the table. The report of the Committee was adopted.

Dr. C. Goodbrake, in behalf of the Nominating Committee, made the following recommendations of committees:—

*For Local Secretary*—Dr. J. Robbins, of Quincy.

*For Committee of Arrangements*—Drs. Louis Watson, J. N. Ralston, and J. T. Wilson, of Quincy; M. Shepherd, of Payson; and W. M. Landon, of Burton.

*For Committee on Necrology*—Drs. J. H. Hollister, of Chicago; F. B. Haller, of Vandalia; and W. S. Edgar, of Jacksonville.

*For Committee on Legislation*—Drs. S. T. Trowbridge, of Decatur; H. A. Johnson, of Chicago; F. B. Haller, of Vandalia; W. S. Edgar, of Jacksonville; and H. Noble, of Heyworth.

*On Cholera-Infantum*—Dr. DeLaskie Miller, of Chicago.

On motion, the report was accepted, and its recommendations adopted.

Dr. H. A. Johnson offered the following, which was adopted:

*Resolved*, That the Committee on Legislation be instructed to prepare and present to the next regular session of the State Legislature, a bill legalizing human dissections, and that each member of this Society be requested to urge upon our legislators the importance of such legal provisions, as a protection to public and private cemeteries, as well as for the promotion of medical and surgical education.

Dr. D. W. Young moved that the thanks of the Society be tendered to Dr. J. Adams Allen, for his interesting public address last evening, and that he be requested to furnish a copy of the same to the Publication Committee, which motion was carried in the affirmative.

Dr. P. Bailhache, Chairman of the Committee on Drugs and Medicines, presented an interesting report, which was accepted and referred to the Committee of Publication.

Dr. David Prince read an abstract of his report on Plastic Surgery, which was accepted, and the report referred to the Committee of Publication.

Dr. H. A. Johnson offered the following, which was adopted:

*Resolved*, That Drs. J. W. Freer and Edmund Andrews be appointed by this Society, delegates to the International Congress, to be held in Paris, during the month of August next.

Dr. E. L. Holmes, Special Committee on Ophthalmology, presented a brief report, which was accepted and referred to the Committee of Publication.

Dr. T. D. Fitch, Chairman of the Committee on Specialties and Medical Advertising, presented a report, which was read by the Secretary and referred to the Committee of Publication.

Dr. D. Prince, from the same committee, presented a minority report, which was also referred to the Committee of Publication.

Dr. J. S. Hildreth read an interesting paper on Granular Conjunctivitis with Paniform Cornea, which was accepted and referred to the Committee of Publication.

Dr. H. A. Johnson presented some interesting illustrations of the pulse lines, as written by the sphygmograph, together with the instrument. The communication was referred to the Publication Committee.

Dr. N. S. Davis read a paper on the Physiological Effects of Alcoholic Drinks, which elicited an interesting discussion, and was referred to the Committee of Publication.

On motion of Dr. D. W. Young, the Society proceeded to elect the following members as delegates to the next annual meeting of the American Medical Association:—

Drs. T. F. Worrell, of Bloomington.
J. H. Hollister, of Chicago.
C. Goodbrake, of Clinton.
Jos. Robbins, of Quincy.
W. S. Edgar, of Jacksonville.
H. B. Buck, of Springfield.
F. B. Haller, of Vandalia.
Moses Gunn, of Chicago.
J. C. Corbus, of LaSalle.
J. T. Wilson, of Quincy.
H. Noble, of Heyworth.
N. Wright, of Chatham.
L. T. Hewins, of Loda.
H. W. Davis, of Paris.
B. K. Shurtleff, of Amboy.
J. M. Steele of Grandview.
E. H. Gale, of Aurora.

Dr. P. H. Bailhache reported that the Treasurer's report has been examined by the Auditing Committee, and found correct.

Dr. J. S. Hildreth offered the following, which was referred to the Committee on Legislation:—

*Resolved*, That a committee of three be appointed, to report at the next annual meeting of this Society, upon the necessity of an act of the Legislature defining the duties, responsibilities, and liabilities of Druggists and Pharmaceutists.

On motion, the Society adjourned, until 7 o'clock P.M.

EVENING SESSION—SECOND DAY.

The Society was called to order by the President at 7 o'clock P.M.

Dr. F. O. Earle, of Chicago, read a paper on the Mechanical Treatment of Angular Curvature of the Spine, and exhibited apparatus. The paper was accepted and referred to the Committee on Publication.

Dr. Addison Niles presented a specimen of Pocket Midwifery Forceps, with a description of the same, which was referred to the Committee of Publication.

Dr. Higgins, of Vandalia, presented the history of a Case of Hydrophobia, which was read by Dr. F. B. Haller, and elicited some discussion. The thanks of the Society were tendered to Dr. Higgins, with the request that the case be published in some medical journal.

Dr. H. A. Johnson offered the following resolution, which was adopted:—

*Resolved*, That this Society urge upon the municipal authorities of all our cities and larger towns the importance of a careful record of births, and a uniform registration of deaths and their causes, using for this purpose such necrological tables as have been generally adopted in this country and Europe.

Dr. D. Prince offered the following, which was adopted:—

*Resolved*, That the Committee on Legislation be instructed to consider the propriety of urging upon the Legislature the passage of a law requiring railroad companies, and other incorporated companies using machinery, to be responsible for the expense of board, nursing, medical supplies, and medical attendance necessary for their employees during the process of recovery, not exceeding six months, in cases of injuries received in the performance of their duties.

The resolution relating to medical education, previously offered by Dr. F. B. Haller, and deferred until the reports of committees, etc., had been disposed of, was now taken up, and after some remarks by Dr. Haller, it was, on motion of Dr. J. S. Whitmire, referred to a committee of three, to report on the same at the next annual meeting of the Society. Drs. F. B. Haller, S. T. Trowbridge, and H. Noble were appointed said committee.

The President also appointed the following committee to examine and report on the status of the Quincy Medical Society: Drs. C. Goodbrake, E. L. Holmes, and D. O. Crist.

The following resolutions were unanimously adopted:—

*Resolved*, That the thanks of this Society be and are hereby tendered to the retiring officers, for their faithful performance of their respective duties.

*Resolved*, That the thanks of this Society are hereby tendered to the profession, citizens, and authorities of Springfield, and also to the Committee of Arrangements, for the provision made for the accommodation and entertainment of the Society at this meeting.

*Resolved*, That the thanks of the Society be tendered to Hon. Sharon Tyndale, the Hon. Secretary of State, for his courtesy in granting the use of the Hall of Representatives for the sessions of the State Medical Society.

*Resolved*, That the thanks of the Society be tendered to the Superintendents of the Illinois Central R.R., the Chicago, Alton and St. Louis R.R., and the Toledo, Wabash, and Great Western R.R., for their favors of commutation tickets to members of the Society attending this meeting.

On motion, the Society adjourned *sine die*.

N. S. DAVIS, *Permanent Sec'y*.
P. H. BAILHACHE, *Ass't-Sec'y*.

